# Supplementary material for: Narrow transmission bottlenecks and limited within-host viral diversity during a SARS-CoV-2 outbreak on a fishing boat
Source: Virus Evol. 2022 Jun 16;8(2):veac052. doi: 10.1093/ve/veac052 (PMC9257191; doi:10.1093/ve/veac052)
Supplement: veac052_Supp [file veac052_supp.zip › Supplemental Figures.docx]

**Supplemental Figures**

**Supplementary Figure 1. The boat was a super spreading event**. (A) We used two standard epidemiological models of outbreaks to simulate an infection on the boat over a range of basic reproduction numbers (R_0_). The gray block highlights a range of R_0_ values for SARS-CoV-2 infection in 2020 from a meta analysis of cohorts (95% CI 2.41, 3.90). The dotted vertical lines designate the R_0_ at which each model recapitulated the percentage of the crew infected on the ship by 16 days. (B) We used a Poisson model from Braun et al. to generate a probability distribution of fixed mutations expected to accumulate over 1, 2, and 3 transmission events (serial intervals). The observed frequency of fixed consensus mutations between the clade containing samples that qualified for resequencing and its inferred ancestral sequence is closest to the distribution of fixed differences from a single transmission event.

**Supplementary Figure 2. Samples were filtered by completeness**. (A) The pattern of sequencing depth for each replicate of all 23 specimens from the boat, and one control sample that was not from the boat (Specimen-10136, labeled with a blue asterisk), that we resequenced for this study. The number of reads per site is capped at 1000X coverage. Samples colored in red have less than 80% of the genome covered by 100X reads. (B) Completeness refers to the percentage of the genome covered by more than 100X reads. Samples colored and highlighted in red have at least one replicate with less than 80% of the genome covered by 100X reads. These samples were excluded from the downstream variant analysis.

**Supplementary Figure 3. Comparison between different variant calling methods**. An UpSet plot shows the overlap in the sets of SNPs called by three different variant calling methods – varscan2, lofreq, ivar, and our custom python script using pysam (*Citations*). Variants were covered by more than 100X reads and present at greater than 2% frequency to be included in the set for each variant caller. The majority of variants are called by all four methods. No variants are called by our custom script that aren’t identified by at least one other method.

**Supplementary Figure 4. Ct value does not correlate with the number of polymorphisms.** Regardless of the variant calling method used, the Ct value of the original nasal swab does not correlate with the number of variants called after filtering out low-frequency (>2%) and poorly covered (>100X) variants. Only samples that passed our quality controls for sequencing completeness (Fig. S3B) and concordance (Fig. 2) were included in this analysis.

**Supplementary Figure 5. There is no discernable pattern of minor variants in the genome.** Plot showing every minor variant (>50% allele frequency) identified across the crew members that passed our quality filters. We included variants if they were present in more than 2% of greater than 100 reads.

**Supplementary Figure 6. Distribution of fixed mutations in the genome.** Plot showing fixed variants identified across the crew members that passed our quality controls. We included variants if they were present in 98% or more of at least 100 reads. Mutations that are present in the 5’ and 3’ UTRs are excluded from this plot.

**Supplementary Figure 7. Low frequency shared variants are present in the non-boat control specimen.** Four variants shared at low-frequency between crew members are also detected in a specimen not collected from the boat but included as a control in both sequencing runs (Specimen 10136). This observation suggests that these are not de novo low-frequency variants that arise on the boat and spread between the crew, but rather sequencing contamination or variant calling errors common to samples from the two sequencing runs.
