# Supplementary figures and images for: Narrow transmission bottlenecks and limited within-host viral diversity during a SARS-CoV-2 outbreak on a fishing boat
Source: Virus Evol. 2022 Jun 16;8(2):veac052. doi: 10.1093/ve/veac052 (PMC9257191; doi:10.1093/ve/veac052)

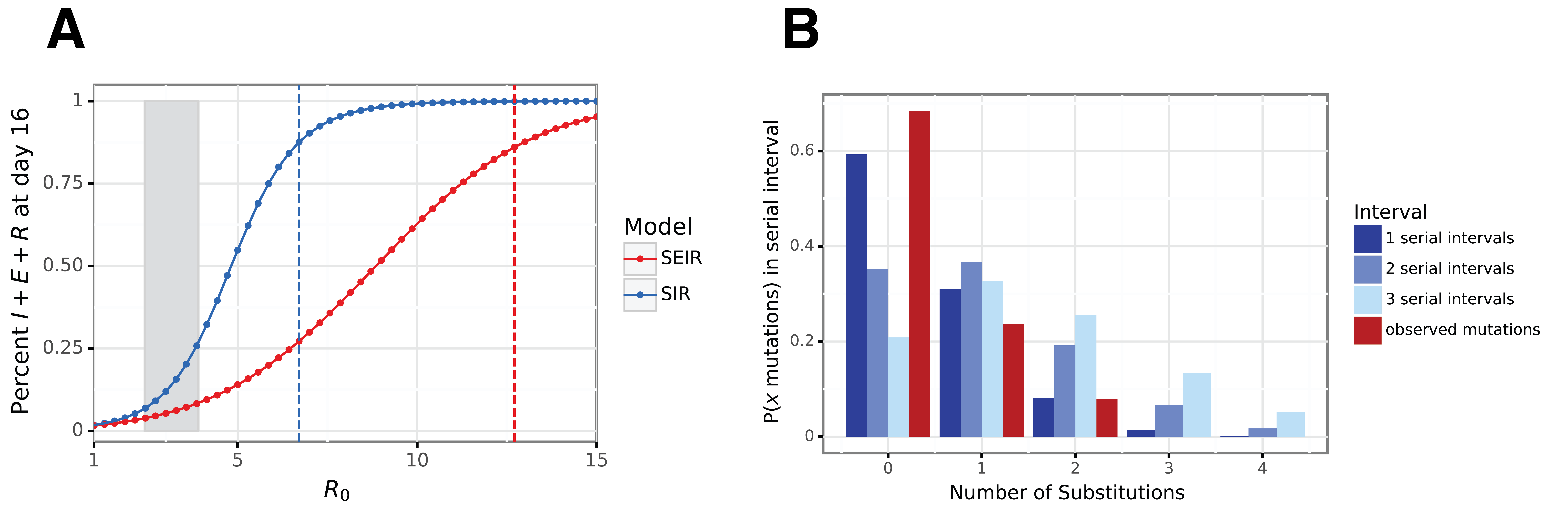

Supplement: veac052_Supp [file veac052_supp.zip › figure-S1.png]

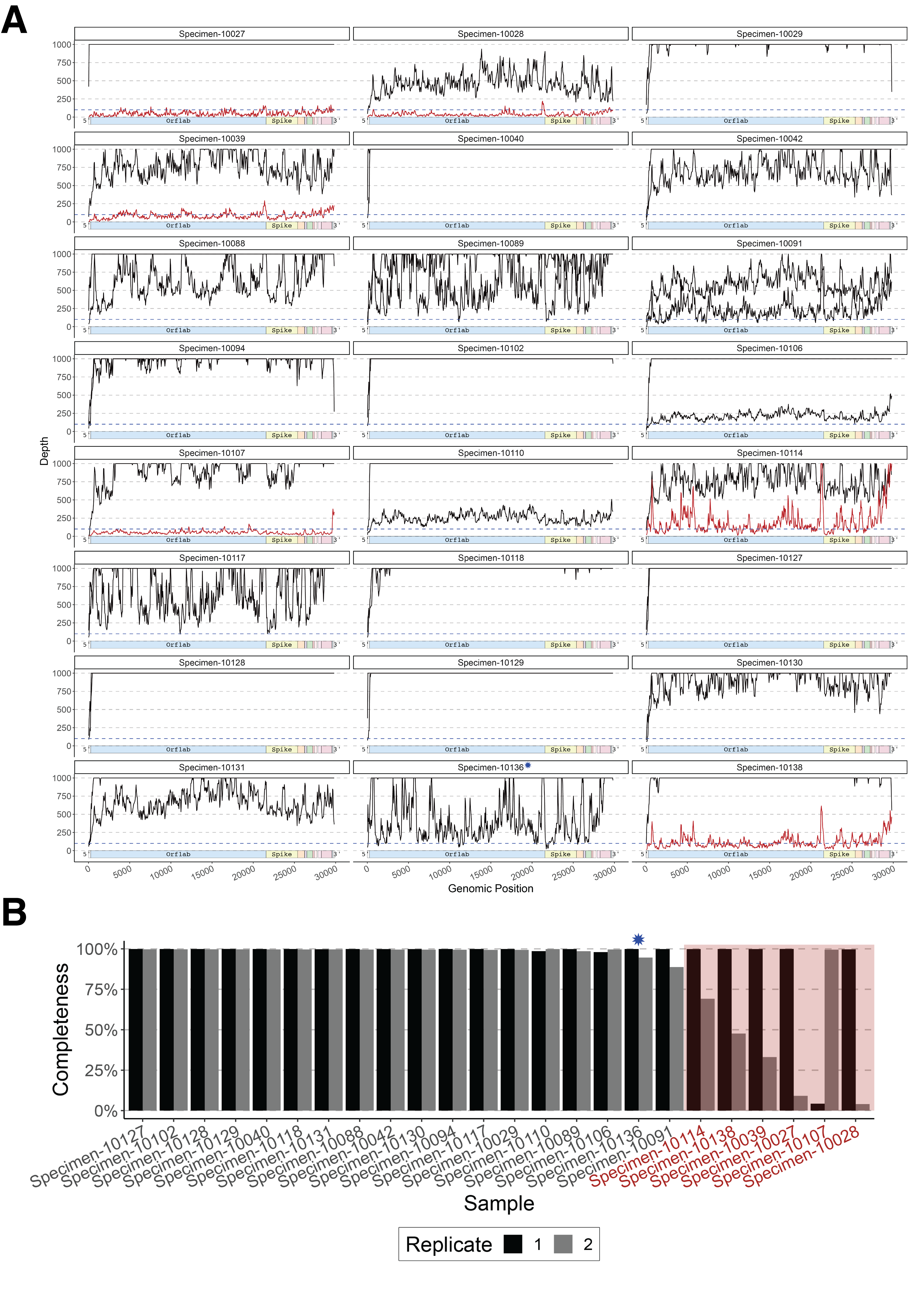

Supplement: veac052_Supp [file veac052_supp.zip › figure-S2.png]

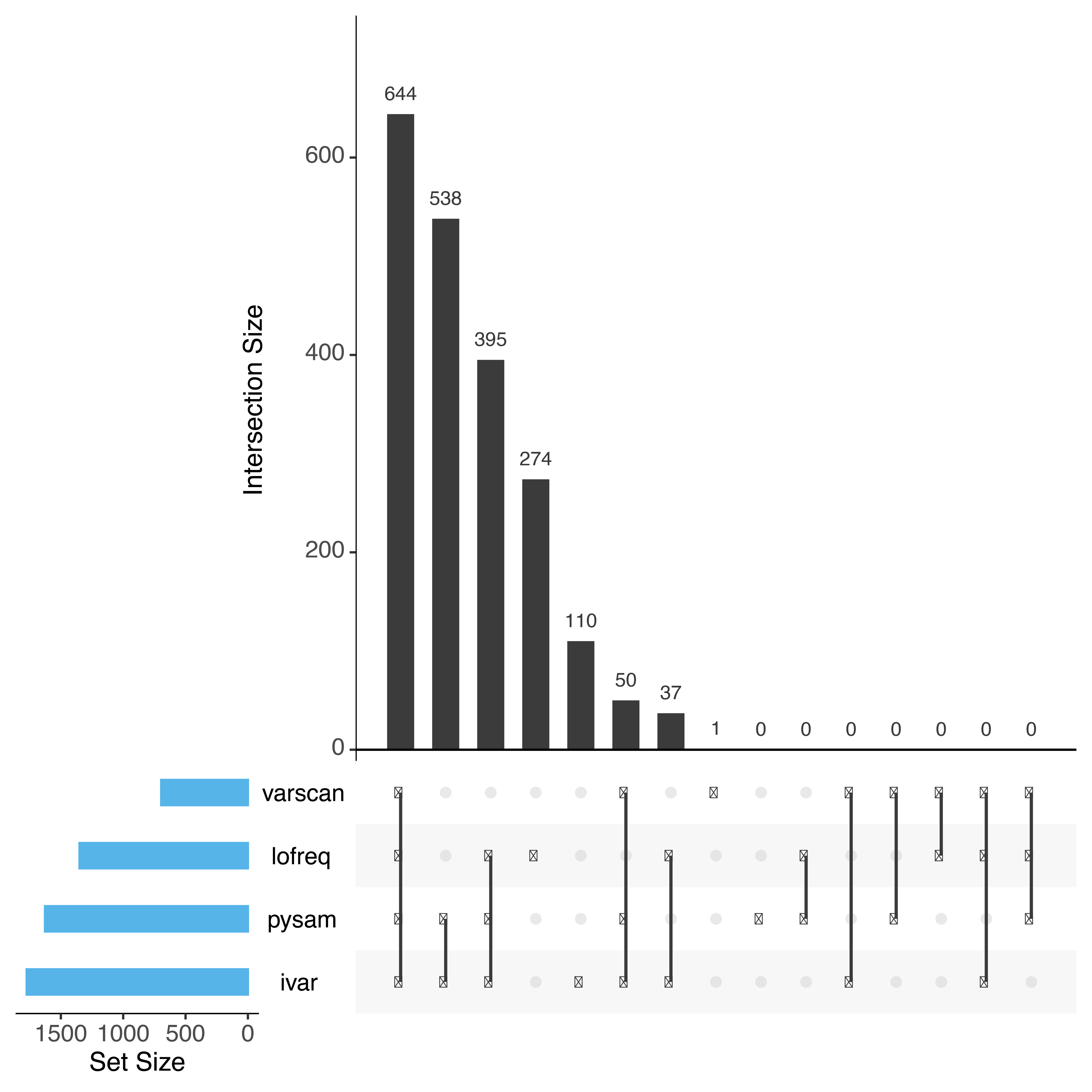

Supplement: veac052_Supp [file veac052_supp.zip › figure-S3.png]

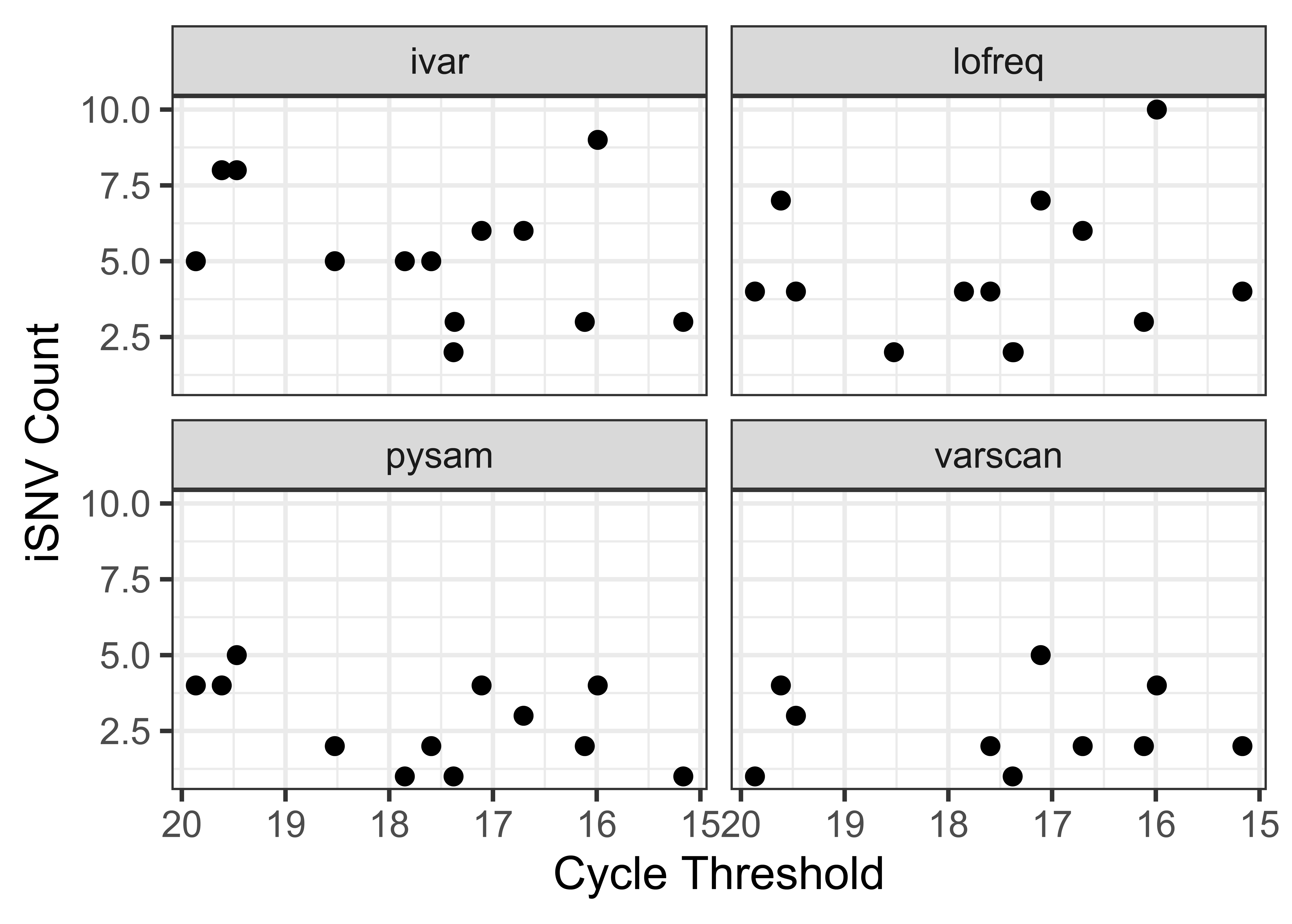

Supplement: veac052_Supp [file veac052_supp.zip › figure-S4.png]

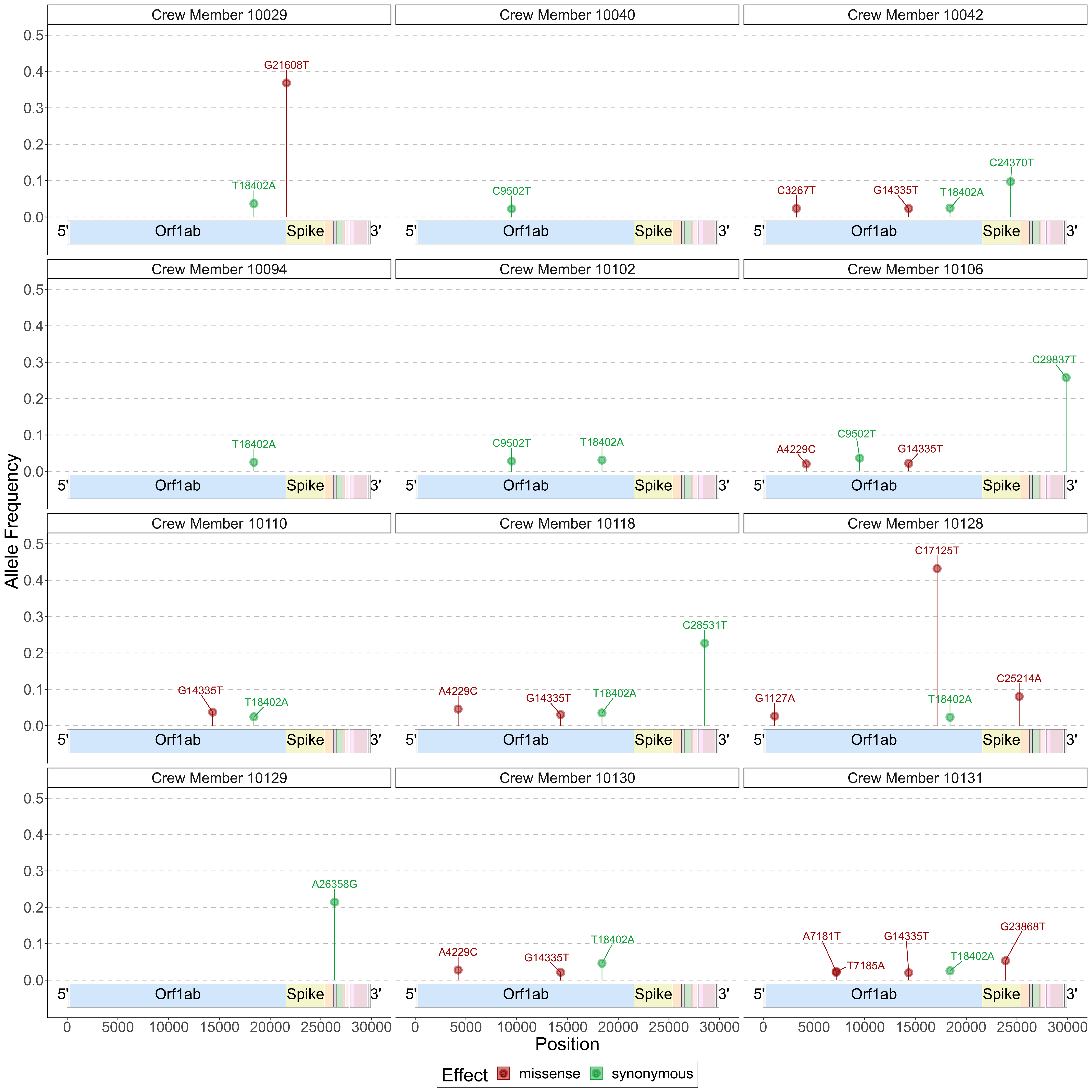

Supplement: veac052_Supp [file veac052_supp.zip › figure-S5.png]

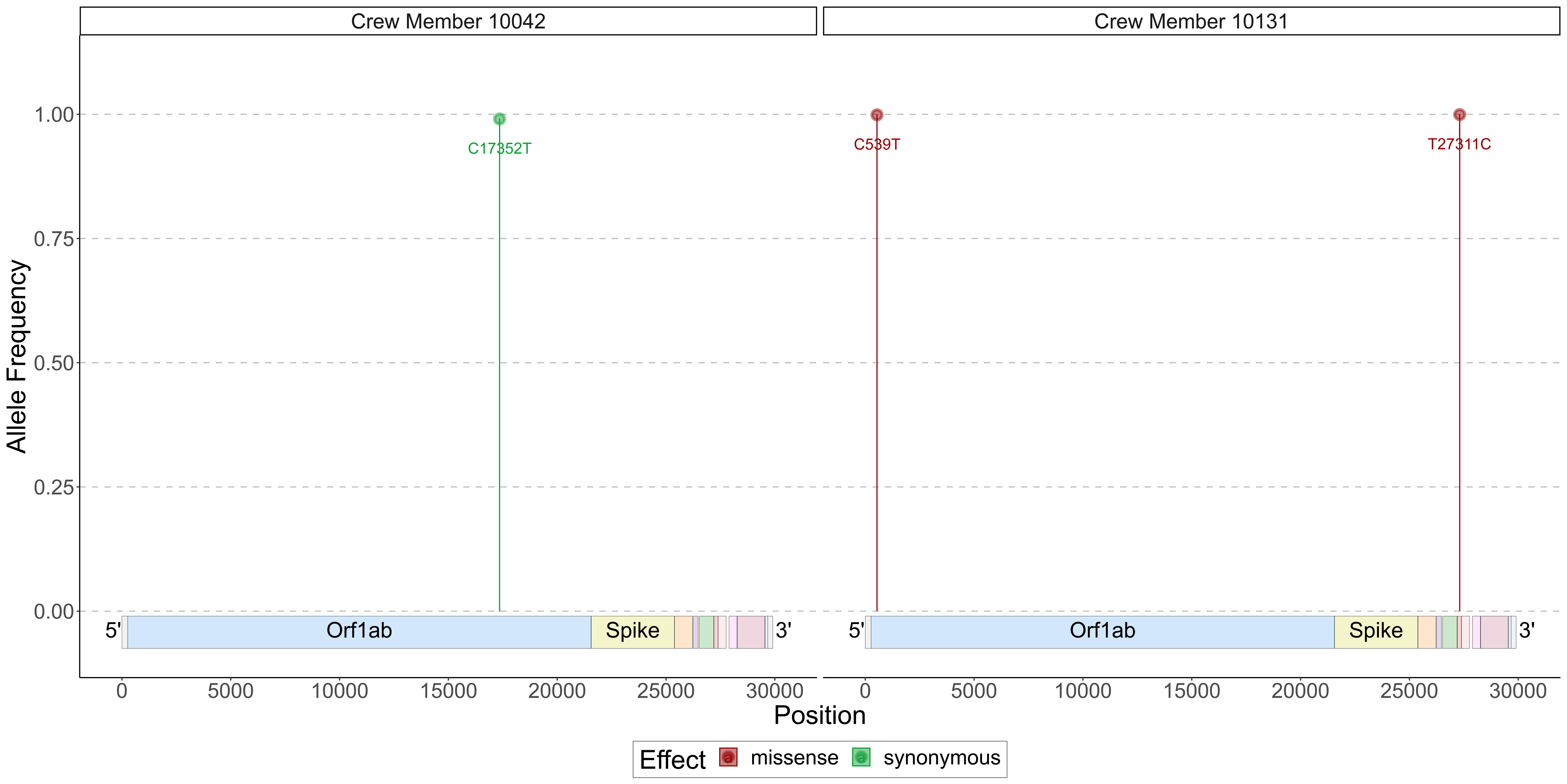

Supplement: veac052_Supp [file veac052_supp.zip › figure-S6.png]

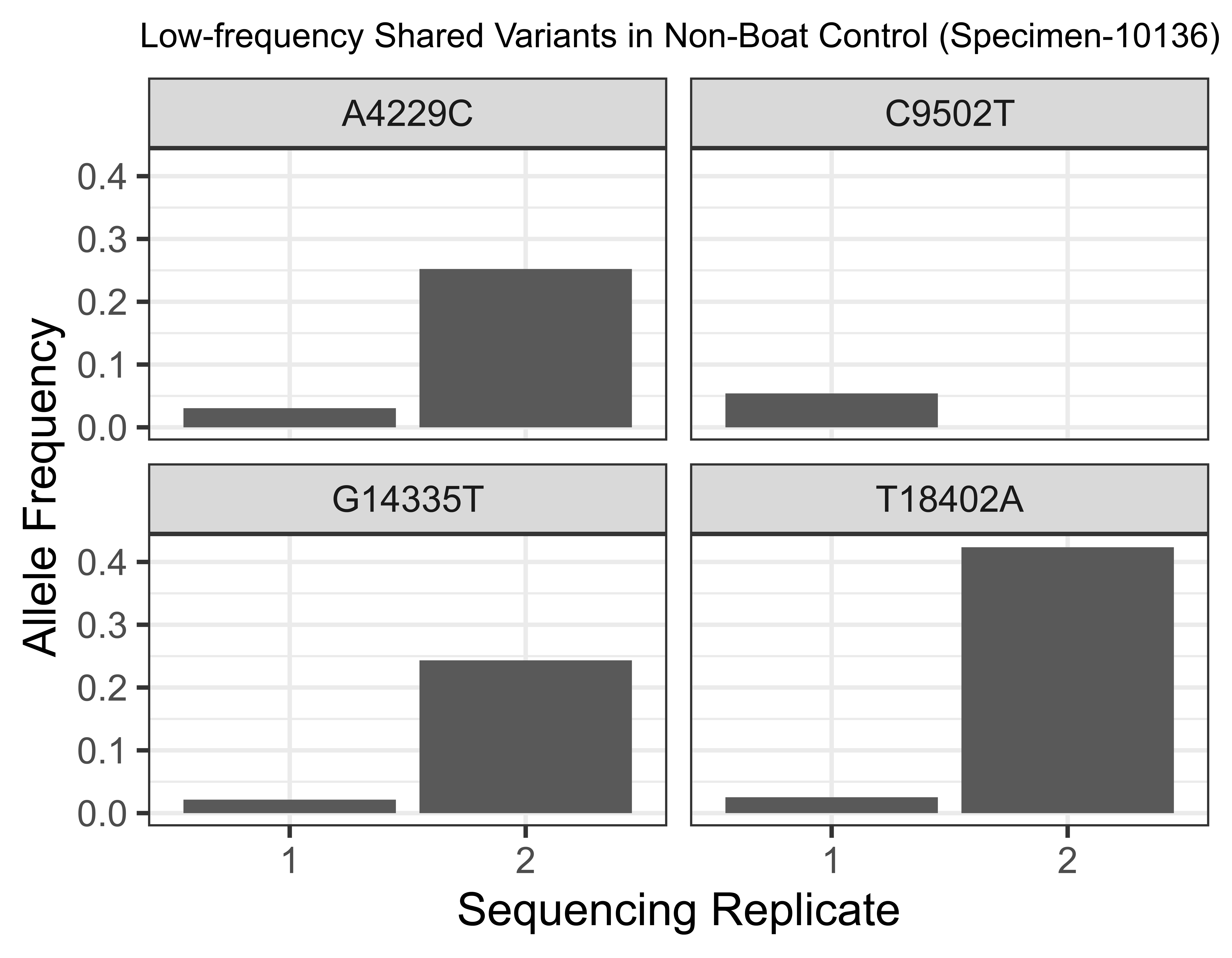

Supplement: veac052_Supp [file veac052_supp.zip › figure-S7.png]
